# 

**Published:** 1879-08

**Authors:** 


					﻿Whole Number
CCCCXI.
August, 1879.
Number 2,
Vol. XXXIX.
THE
CHICAGO
MEDICAL
T ournal and Examiner
EDITORS:
WILLIAM H. BYFORD, A.M.,'M.D.,
JAS. NEVINS HYDE, A.M., M.D. N. S. DAVIS, A.M., M.D.
IDANIEL R. BROWER, M.D.
CHICAGO:
PUBLISHED BY THE CHICAGO MEDICAL PRESS ASSOCIATION.
188 Clark Street.
®"Read the Notice of this. Journal among Advertisements.
				

